# Synergistic induction of cancer-related immunosuppression by β2-adrenergic stress mediators and P38MAPK inflammatory pathway

**DOI:** 10.1186/2051-1426-1-S1-P191

**Published:** 2013-11-07

**Authors:** Ravikumar Muthuswamy, Frank Jenkins, Dana Bovbjerg, Pawel Kalinski

**Affiliations:** 1 Surgery, University of Pittsburgh, Pittsburgh, PA, USA; 2 Pathology, University of Pittsburgh, Pittsburgh, PA, USA; 3 Psychiatry, University of Pittsburgh, Pittsburgh, PA, USA; 4 Immunology, University of Pittsburgh, Pittsburgh, PA, USA

## Background

Psychological stress has been implicated in promoting cancer recurrence and progression, but the magnitude of this effect and underlying mechanism remain unclear. In attempt to address this controversy, we have evaluated the impact of stress on molecular and cellular components of cancer Immune-surveillance and tested the hypothesis that the impact of stress in promoting tumor-associated immune suppression is context-dependent.

## Materials and methods

Modulatory impact of epinephrine (EPI) was tested ex vivo, in cultured myeloid cells, CD4+ or CD8+ T cells or while tumor tissue explant cultures. Celecoxib (COX-2 inhibitor) was used to assess whether EPI mediated generation of MDSC/Treg involves COX-2 induction. In order to delineate the specific signaling pathway involved in EPI mediated induction of suppressive markers in MDSC, Treg and tumor explant cultures, selective inhibitors of PKA(H89), PKB(MK2206) and p38 MAPK(PH797804) were used.

## Results

Epinephrine showed strong synergy with TNFα and IL1mediators of tumor-associated inflammation, in the induction of high levels of mediators of tumor-associated immune suppression (COX-2, IDO) and Treg-attracting chemokine, CCL22. Epinephrine-driven immunosuppressive events were highly dependent on the availability of TNFα or IL1-dirven inflammation. Macrophages cultured in presence of TNFα + EPI suppressed CD3/CD28 stimulated proliferation of naïve CD8+ T cells. CD4+ T cells showed up regulation of suppressive mediator, IL-10, reduced proliferation and reduced IFNγ expression when stimulated in presence of epinephrine. Suppressive effects of EPI were antagonized by celecoxib, and blocked by the combination of propranolol (β2-adrenergic antagonist) and celecoxib (COX-2 blocker) or by inhibitors of PKA and p38 MAPK pathways.

## Conclusions

Current demonstration that immunosuppressive effects of stress mediators are context-dependent and require the component of p38MAPK-mediated inflammation helps to explain current controversy regarding the link between psychological stress and cancer recurrence/progression. That interplay between stress, inflammation and COX-2/PGE2 system in the development of tumor-associated immunosuppression suggests the possibility of new combination immunotherapies of cancer, involving β2-adrenergic antagonists, COX2 blockers and/or or p38MAPK inhibitors.

